# Outcomes after distal pancreatectomy with or without splenectomy for intraductal papillary mucinous neoplasm: international multicentre cohort study

**DOI:** 10.1093/bjs/znad424

**Published:** 2024-01-09

**Authors:** Myrte Gorris, Eduard A van Bodegraven, Mohammad Abu Hilal, Louisa Bolm, Olivier R Busch, Marco del Chiaro, Joseph Habib, Kiyoshi Hasegawa, Jin He, Jeanin E van Hooft, Jin-Young Jang, Ammar A Javed, Yusuke Kazami, Wooil Kwon, Mirang Lee, Rong Liu, Fuyuhiko Motoi, Giampaolo Perri, Akio Saiura, Roberto Salvia, Hideki Sasanuma, Yoshinori Takeda, Christopher Wolfgang, Piotr Zelga, Carlos Fernandez - del Castillo, Giovanni Marchegiani, Marc G Besselink

**Affiliations:** Department of Surgery, Amsterdam UMC, Location University of Amsterdam, Amsterdam, the Netherlands; Department of Gastroenterology and Hepatology, Amsterdam UMC, location University of Amsterdam, Amsterdam, the Netherlands; Amsterdam Gastroenterology Endocrinology Metabolism, Amsterdam, the Netherlands; Cancer Centre Amsterdam, Amsterdam, the Netherlands; Department of Surgery, Amsterdam UMC, Location University of Amsterdam, Amsterdam, the Netherlands; Amsterdam Gastroenterology Endocrinology Metabolism, Amsterdam, the Netherlands; Cancer Centre Amsterdam, Amsterdam, the Netherlands; Department of Hepatopancreatobiliary Surgery, University Hospital Southampton, Southampton, UK; Department of Surgery, Foundation Poliambulanza, Brescia, Italy; Department of Surgery, Massachusetts General Hospital, Harvard Medical School, Boston, Massachusetts, USA; Department of Surgery, Amsterdam UMC, Location University of Amsterdam, Amsterdam, the Netherlands; Amsterdam Gastroenterology Endocrinology Metabolism, Amsterdam, the Netherlands; Cancer Centre Amsterdam, Amsterdam, the Netherlands; Department of Surgery, University of Colorado Anschutz Medical Campus, Aurora, Colorado, USA; Department of Surgery, Johns Hopkins University School of Medicine, Baltimore, Maryland, USA; Hepato-Biliary-Pancreatic Surgery Division, Department of Surgery, Graduate School of Medicine, University of Tokyo, Tokyo, Japan; Department of Surgery, Johns Hopkins University School of Medicine, Baltimore, Maryland, USA; Department of Gastroenterology and Hepatology, Leiden University Medical Centre, Leiden, the Netherlands; Department of Surgery and Cancer Research Institute, Seoul National University College of Medicine, Seoul, Korea; Department of Surgery, Johns Hopkins University School of Medicine, Baltimore, Maryland, USA; Hepato-Biliary-Pancreatic Surgery Division, Department of Surgery, Graduate School of Medicine, University of Tokyo, Tokyo, Japan; Department of Surgery and Cancer Research Institute, Seoul National University College of Medicine, Seoul, Korea; Department of Surgery and Cancer Research Institute, Seoul National University College of Medicine, Seoul, Korea; Faculty of Hepatopancreatobiliary Surgery, First Medical Centre of Chinese People’s Liberation Army (PLA) General Hospital, Beijing, China; Department of Surgery I, Yamagata University, Yamagata, Japan; Department of General and Pancreatic Surgery, Verona University Hospital, Verona, Italy; Department of Hepatobiliary–Pancreatic Surgery, Juntendo University School of Medicine, Hongo, Tokyo, Japan; Department of General and Pancreatic Surgery, Verona University Hospital, Verona, Italy; Department of Surgery, Jichi Medical University, Shimotsuke, Tochigi, Japan; Department of Hepatobiliary–Pancreatic Surgery, Juntendo University School of Medicine, Hongo, Tokyo, Japan; Department of Surgery, NYU Grossman School of Medicine, NewYork, New York, USA; Department of Surgery, Massachusetts General Hospital, Harvard Medical School, Boston, Massachusetts, USA; Department of Surgery, Massachusetts General Hospital, Harvard Medical School, Boston, Massachusetts, USA; Department of General and Pancreatic Surgery, Verona University Hospital, Verona, Italy; Department of Surgery, Amsterdam UMC, Location University of Amsterdam, Amsterdam, the Netherlands; Amsterdam Gastroenterology Endocrinology Metabolism, Amsterdam, the Netherlands; Cancer Centre Amsterdam, Amsterdam, the Netherlands

## Abstract

**Background:**

International guidelines on intraductal papillary mucinous neoplasm (IPMN) recommend a formal oncological resection including splenectomy when distal pancreatectomy is indicated. This study aimed to compare oncological and surgical outcomes after distal pancreatectomy with or without splenectomy in patients with presumed IPMN.

**Methods:**

An international, retrospective cohort study was undertaken in 14 high-volume centres from 7 countries including consecutive patients after distal pancreatectomy for IPMN (2005–2019). Patients were divided into spleen-preserving distal pancreatectomy (SPDP) and distal pancreatectomy with splenectomy (DPS). The primary outcome was lymph node metastasis (LNM). Secondary outcomes were overall survival, duration of operation, blood loss, and secondary splenectomy.

**Results:**

Overall, 700 patients were included after distal pancreatectomy for IPMN; 123 underwent SPDP (17.6%) and 577 DPS (82.4%). The rate of malignancy was 29.6% (137 patients) and the overall rate of LNM 6.7% (47 patients). Patients with preoperative suspicion of malignancy had a LNM rate of 17.2% (23 of 134) *versus* 4.3% (23 of 539) among patients without suspected malignancy (*P* < 0.001). Overall, SPDP was associated with a shorter operating time (median 180 *versus* 226 min; *P* = 0.001), less blood loss (100 *versus* 336 ml; *P* = 0.001), and shorter hospital stay (5 *versus* 8 days; *P* < 0.001). No significant difference in overall survival was observed between SPDP and DPS for IPMN after correction for prognostic factors (HR 0.50, 95% c.i. 0.22 to 1.18; *P* = 0.504).

**Conclusion:**

This international cohort study found LNM in 6.7% of patients undergoing distal pancreatectomy for IPMN. In patients without preoperative suspicion of malignancy, SPDP seemed oncologically safe and was associated with improved short-term outcomes compared with DPS.

## Teaser

Guidelines recommend splenectomy in patients requiring distal pancreatectomy for intraductal papillary mucinous neoplasm (IPMN) as the safety of spleen-preserving distal pancreatectomy (SPDP) is unknown. Among 700 distal pancreatectomies for IPMN, lymph node metastases were present in 6.7% (47 patients) *versus* 4.3% of those without suspected malignancy. In patients without preoperative suspicion of malignancy, SPDP seemed oncologically safe and was associated with improved short-term outcomes.

## Introduction

Pancreatic cystic neoplasms are being detected at an increasing rate because of the expanding use of high-quality cross-sectional imaging^1,2^. A weighted incidence of incidental pancreatic cysts of up to 49% has been reported in the general population^3^. The most common pancreatic cystic neoplasm is intraductal pancreatic mucinous neoplasm (IPMN), for which surveillance is mostly recommended, whereas high-risk patients (for example those with IPMN with mural nodules, jaundice, and main duct dilatation exceeding 10 mm) are recommended to undergo resection to prevent malignant degeneration^4,5^.

Distal pancreatectomy is the standard surgical procedure for IPMN located in the pancreatic body and tail requiring resection. In patients with malignant disease (such as pancreatic cancer), distal pancreatectomy is routinely combined with splenectomy to ensure radical resection of potential lymph node metastases (LNMs). At present, both international^4^ and European^5^ guidelines recommend distal pancreatectomy with splenectomy for all patients with IPMN requiring distal pancreatectomy. However, the need for distal pancreatectomy with splenectomy in patients with premalignant IPMN remains unclear because this advice is based on small cohort studies, and a possible survival benefit compared with spleen-preserving distal pancreatectomy has never been proven.

Splenectomy has been associated with an impaired immune response, need for immunization, and a 0.1–8.5% risk of a potentially lethal overwhelming postsplenectomy infection (OPSI)^6^. Furthermore, long-term follow-up studies in American veterans^7,8^ have shown an increased risk of death from pneumonia, ischaemic heart disease, septicaemia, pulmonary embolism, and different types of cancer, even more than 10 years after splenectomy. Patients with resected IPMN have an excellent prognosis (pooled 5-year survival rate 93.6% in 2868 patients)^9^ and could therefore benefit from spleen preservation. In general, spleen-preserving distal pancreatectomy has been associated with less blood loss, shorter hospital stay and improved long-term health outcomes compared with distal pancreatectomy with splenectomy^10,11^.

This study aimed to assess the oncological and surgical outcomes of spleen-preserving distal pancreatectomy and distal pancreatectomy with splenectomy in patients with presumed IPMN with and without suspected malignancy in a large, international, multicentre cohort. The primary outcome was the rate of LNM. Secondary outcomes included overall survival (OS), duration of operation, estimated blood loss, and need for secondary splenectomy.

## Methods

### Study design

This was an international, multicentre retrospective cohort study that included centres participating in the Verona Evidence Based Medicine (EBM) 2020 IPMN consortium. The present manuscript was redacted and drafted under the auspices of this consortium^12^. Patients were included from 14 high-volume centres (defined by at least 15 distal pancreatectomies per year for all indications) in 7 countries, which all performed distal pancreatectomy with splenectomy and spleen-preserving distal pancreatectomy. This study was conducted in accordance with the STROBE guidelines for reporting observational studies^13^. The study protocol was approved by the institutional review board of Amsterdam UMC and the requirement to obtain informed consent was waived. All participating institutions followed local regulations regarding study approval and informed consent procedures.

### Study population

Consecutive patients who had undergone distal pancreatectomy, either spleen-preserving distal pancreatectomy or distal pancreatectomy with splenectomy, for presumed IPMN between 1 January 2005 and 31 December 2019 were eligible for inclusion. For spleen-preserving distal pancreatectomy, both the Warshaw procedure (splenic vessel resecting)^14^ and the Kimura procedure (splenic vessel preserving)^15^ were included. Planned distal pancreatectomy with splenectomy included patients in whom splenectomy was planned before operation, thereby excluding emergency splenectomies. Patients were excluded if essential information was lacking (surgical or pathology reports missing) or if pancreatic resections other than distal pancreatectomy were performed. The diagnosis of IPMN was based on the preoperative assessment by the local multidisciplinary team. Subgroup analyses were undertaken for patients with and without preoperative suspicion of malignancy. Patients were classified as having suspected malignancy if there was preoperative suspicion of a solid mass, cytology showing malignancy, or lymphadenopathy on preoperative imaging. All other patients were classified as not having a suspected malignancy, regardless of the postoperative dysplasia grade. Patients in whom the indication for resection was unknown were omitted from these subgroup analyses.

### Data collection

Invitations to participate in the present study were distributed via e-mail through the EBM 2020 on IPMN consortium. After an initial participation survey (Google™ Survey, Mountain View, CA, USA) confirming the study requirements, each participating centre appointed one dedicated local study coordinator, who was responsible for all communication with the central study coordinator. The local study coordinator was responsible for data entry into the electronic case report form using Castor EDC^16^. An overview of the variables collected is available in *Table S1*.

### Outcomes

The primary outcome was the rate of LNM, both overall and in patients with and those without suspected malignancy. Secondary outcomes included duration of operation, estimated blood loss, 90-day pancreas-specific complications, major in-hospital morbidity (Clavien–Dindo grade IIIa or higher)^17^, long-term postoperative morbidity (new-onset diabetes mellitus, exocrine pancreatic insufficiency, and secondary splenectomy), and OS. OS was defined as the interval between the date of surgery and date of death or last follow-up. The definitions of the International Study Group on Pancreatic Surgery were used to score postoperative pancreatic fistula^18^, delayed gastric emptying^19^, chyle leak^20^, and postpancreatectomy haemorrhage^21^. Only grade B/C complications were included. Ischaemic morbidity was defined as an abdominal organ complication caused by surgery-related ischaemia. Lymph node stations were reported according to the Japanese classification of pancreatic cancer^22^. Disease staging was carried out according to the seventh version of the AJCC TNM classification^23^ until 2017; the eighth version of the AJCC^24^ was used from 2018 onwards.

### Statistical analysis

Categorical data are presented as numbers with percentages, and were analysed using the χ^2^ test or Fisher’s exact test, if appropriate. Continuous data are presented as median (i.q.r.) and were compared using the Mann–Whitney *U* test. OS was calculated using the Kaplan–Meier method and analysed using the log rank test.

All *P* values were based on a two-sided test and *P* < 0.050 was considered statistically significant. Data were analysed with the use of SPSS^®^ Statistics for Windows^®^ version 26.0 (IBM, Armonk, NY, USA).

## Results

**Fig. 1:**
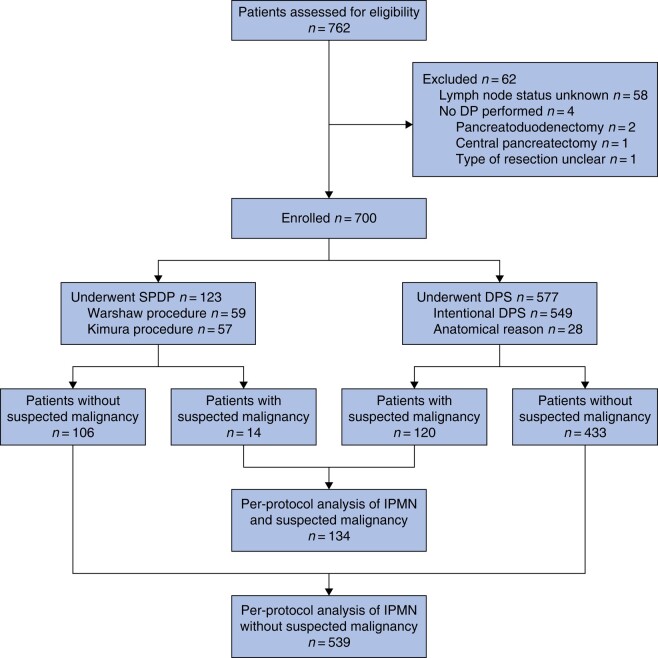
Study flow chart DP, distal pancreatectomy; SPDP, spleen-preserving distal pancreatectomy; DPS, distal pancreatectomy with splenectomy; IPMN, intraductal papillary mucinous neoplasm.

Overall, 700 patients were included, of whom 123 (17.6%) had undergone spleen-preserving distal pancreatectomy and 577 (82.4%) distal pancreatectomy with splenectomy (*Fig. 1*). The majority of these patients underwent planned splenectomy (549 patients, 95.1%). Spleen-preserving distal pancreatectomy was performed by the Warshaw procedure in 59 patients (48.0%) and the Kimura procedure in 57 (46.3%); the procedure type was unknown for 7 patients (5.7%). All but one hospital performed both spleen-preserving distal pancreatectomy and distal pancreatectomy with splenectomy, and one hospital performed only distal pancreatectomy with splenectomy. One hospital carried out more spleen-preserving distal pancreatectomies than distal pancreatectomies with splenectomy. The cohort had a median age of 70 (i.q.r. 63–76) years and 344 patients (49.1%) were men. Patients undergoing spleen-preserving distal pancreatectomy were younger (68 (61–74) *versus* 70 (63–76) years; *P* = 0.003) (*Table 1*). The most common indication for resection was a dilated main pancreatic duct, which was present in 329 patients (47.0%) in the total cohort. An overview of all indications for resection is available in *Table S2*. Most patients were diagnosed with low-grade dysplasia (242, 34.6%), whereas 165 (23.6%) had intermediate-grade dysplasia, 120 (17.1%) had high-grade dysplasia, and 137 (19.6%) had invasive cancer in IPMN. In the total cohort, a median of 11 (i.q.r. 5–20) lymph nodes were harvested per patient and 47 patients (6.7%) had LNM.

**Table 1:** Baseline characteristics of 700 patients after distal pancreatectomy for suspected intraductal papillary mucinous neoplasm

	SPDP (*n* = 123)	DPS (*n* = 577)	*P*‡
**Sex**			0.494
Male	57 (46.3)	287 (49.7)	
Female	66 (53.7)	290 (50.3)	
**Age (years), median (i.q.r.)**	68 (61–74)	70 (63–76)	0.003§
**WHO performance status**			0.981
0–1	104 (84.5)	481 (83.4)	
2–4	9 (7.3)	42 (7.3)	
Missing	10 (8.1)	54 (9.4)	
**ASA fitness grade**			0.243
I–II	95 (77.2)	400 (69.3)	
III–IV	28 (22.7)	155 (26.9)	
Missing	0 (0)	22 (3.8)	
**History of pancreatitis**	22 (17.9)	99 (17.2)	0.853
**Co-morbidities**	95 (77.2)	428 (74.2)	0.479
Cardiac	34 (27.6)	138 (23.9)	
Vascular	28 (22.7)	184 (31.9)	
Diabetes	15 (12.2)	128 (22.2)	
Pulmonary	11 (8.9)	57 (9.9)	
Neurological	4 (3.2)	18 (3.1)	
Gastrointestinal	31 (25.2)	78 (13.5)	
Urogenital	5 (4.1)	15 (2.6)	
Renal	6 (4.9)	26 (4.5)	
Connective tissue disease	0 (0)	6 (1.0)	
Immunological	3 (2.4)	14 (2.4)	
Oncological	23 (18.7)	75 (13.0)	
Other	5 (4.1)*	13 (2.2)†	

Values are *n* (%) unless otherwise indicated. Other co-morbidities included *hyperthyroidism (2), hypothyroidism (2), and haemochromatosis (1); †hyperthyroidism (1), hypothyroidism (8), gout (2), Cushing syndrome (1), and Addison disease (1). SPDP, spleen-preserving distal pancreatectomy; DPS, distal pancreatectomy with splenectomy. ‡χ^2^ test or Fisher’s exact test, except §Mann–Whitney *U* test.

### Per-protocol analysis

In the per-protocol analysis of the subgroup of 134 patients with IPMN and suspected malignancy, 74 (55.2%) were diagnosed with invasive cancer. This included 4 of 14 patients (29%) after spleen-preserving distal pancreatectomy and 70 of 120 (58.3%) after distal pancreatectomy with splenectomy (*P* = 0.104 overall) (*Table S3*). LNMs were found in 1 of 14 patients (7%) after spleen-preserving distal pancreatectomy and 22 of 120 (18.3%) after distal pancreatectomy with splenectomy (relative risk (RR) 0.39, 95% c.i. 0.06 to 2.67; *P* = 0.463).

In the per-protocol analysis of the subgroup of 539 patients without suspected malignancy, 61 patients (11.3%) were diagnosed with invasive cancer. These included 5 patients (5%) after spleen-preserving distal pancreatectomy and 56 (13%) after distal pancreatectomy with splenectomy (*P* < 0.001 overall) (*Table 2*). Fewer lymph nodes were harvested in patients undergoing spleen-preserving distal pancreatectomy (median 4 (i.q.r. 1–7) *versus* 12 (5–20); *P* < 0.001). In total, LNMs were found in 23 of 539 patients (4.3%). Among those without suspected malignancy, LNMs were found in 1 of 106 patients (0.9%) who had spleen-preserving distal pancreatectomy, compared with 22 of 433 (5.1%) after distal pancreatectomy with splenectomy (RR 0.19, 0.03 to 1.36; *P* = 0.062).

**Table 2:** Pathological outcome and lymph node metastases in the per-protocol analysis of 539 patients with intraductal papillary mucinous neoplasm after distal pancreatectomy without preoperative suspicion of malignancy

	SPDP (*n* = 106)	DPS (*n* = 433)	Relative risk*	*P*‡
**Grade of dysplasia**			–	< 0.001§
Low	57 (53.8)	150 (34.6)		
Intermediate	24 (22.6)	123 (28.4)		
High	12 (11.3)	88 (20.3)		
Invasive cancer	5 (4.7)	56 (12.9)		
Not reported	8 (7.5)	16 (3.7)		
No. of lymph nodes harvested, median (i.q.r.)	4 (1–7)	12 (5–20)	–	< 0.001¶
**No. of patients with positive lymph nodes**	1 of 106 (0.9)	22 of 433 (5.1)	0.19 (0.03, 1.36)	0.062
Nodule or enhancing wall	1 of 19 (5)	5 of 97 (5)	1.02 (0.13, 8.26)	> 0.99
Main duct involvement	0 of 43 (0)	10 of 184 (5)	–	0.215
Growth or cyst size	0 of 37 (0)	5 of 104 (5)	–	0.326
Increased CA19.9 level	0 of 1 (0)	1 of 6 (17)	–	> 0.99
Clinical symptoms†	0 of 4 (0)	0 of 6 (0)	–	–
Other indication	0 of 2 (0)	1 of 12 (8)	–	> 0.99

Values are *n* (%) unless otherwise indicated: *values in parentheses are 95% confidence intervals. †New onset or worsening of pre-existing diabetes mellitus, pancreatitis, or persisting abdominal symptoms. SPDP, spleen-preserving distal pancreatectomy; DPS, distal pancreatectomy with splenectomy; CA19.9, carbohydrate antigen 19.9. ‡Fisher’s exact test, except §χ^2^ test and ¶Mann–Whitney *U* test.

### Intention-to-treat analysis

Intention-to-treat analysis of the subgroup of patients with a preoperative suspicion of malignancy yielded comparable results (*Table S4*). In intention-to-treat analysis of patients without suspected malignancy, similar results were observed (*Table S5*).

### Surgical outcomes

In the overall cohort, spleen-preserving distal pancreatectomy was more frequently performed with the use of minimally invasive surgery (59 of 123, 48.0%) than distal pancreatectomy with splenectomy (162 of 577, 28.1%) (*P* < 0.001) (*Table S6*). Operating time was 46 min shorter in patients undergoing spleen-preserving distal pancreatectomy (median 180 (i.q.r. 120–241) *versus* 226 (162–280) min; *P* = 0.001), and blood loss was 236 ml less with spleen preservation (100 (50–250) *versus* 336 (100–383) ml; *P* = 0.001). Hospital stay was 3 days shorter in patients who underwent spleen-preserving distal pancreatectomy (median 5 (i.q.r. 4–7) *versus* 8 (6–14) days). None of the patients needed a secondary splenectomy after an initial spleen-preserving distal pancreatectomy. There was no difference in adverse events after spleen-preserving distal pancreatectomy *versus* distal pancreatectomy with splenectomy. New-onset diabetes mellitus occurred in 175 patients (25.0%) and exocrine pancreatic insufficiency in 114 (16.3%).

### Overall survival

After a median follow-up of 52 months, 115 of 700 patients (16.4%) had died. Mean estimated survival time was better after spleen-preserving distal pancreatectomy (154 (95% c.i. 144 to 164) months) than after distal pancreatectomy with splenectomy (145 (137 to 153) months) (*P* = 0.005). The 1-, 3- and 5-year estimated survival rates were, respectively, 99, 97, and 95% in patients with low-grade dysplasia, 96, 92, and 89% for those with intermediate-grade dysplasia, and 96, 89, and 77% for patients with high-grade dysplasia. Estimated survival rates for those with invasive cancers were 87% after 1 year, 66% after 3 years, and 53% after 5 years.

**Fig. 2:**
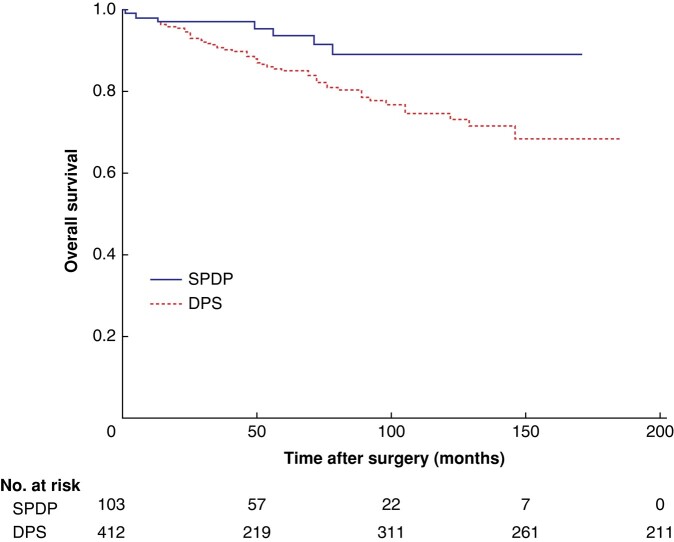
Kaplan–Meier estimates of overall survival after spleen-preserving distal pancreatectomy *versus* distal pancreatectomy with splenectomy in patients without suspected malignancy SPDP, spleen-preserving distal pancreatectomy; DPS, distal pancreatectomy with splenectomy.

Among patients without suspected malignancy, a total of 73 of 539 (13.5%) had died after a median follow-up of 55 months. Of these, 7 (7%) died after spleen-preserving distal pancreatectomy *versus* 66 (15%) after distal pancreatectomy with splenectomy. Mean estimated OS was better after spleen-preserving distal pancreatectomy (158 (148 to 167) *versus* 152 (143 to 160) months; *P* = 0.017) (*Fig. 2*). However, the association between spleen-preserving distal pancreatectomy and OS did not remain after correction for dysplasia grade (HR 0.48, 95% c.i. 0.29 to 1.11; *P* = 0.085) (*Fig. S1*). The result was similar when both dysplasia grade and age were corrected for in multivariable analysis (HR 0.50, 0.22 to 1.18; *P* = 0.504) (*Table 3*). Factors associated with worse OS were age (HR 1.95, 1.20 to 3.12; *P* = 0.007), high-grade dysplasia (HR 2.96, 1.39 to 6.29; *P* = 0.005), and invasive cancer (HR 8.26, 3.95 to 17.27; *P* < 0.001).

**Table 3:** Cox regression analyses evaluating predictors of overall survival in patients without suspected malignancy

	Univariate analysis		Multivariable analysis	
	HR	*P*	HR	*P*
Age > 70 years	1.96 (1.22, 3.25)	0.006	1.95 (1.20, 3.12)	0.007
SPDP	0.41 (0.21, 0.79)	0.007	0.50 (0.22, 1.18)	0.504
Nodule or enhancing wall	1.44 (0.86, 2.43)	0.165	–	–
Main duct involvement	0.75 (0.46, 1.23)	0.251	–	–
Growth or cyst size	0.95 (0.56, 1.61)	0.847	–	–
Increased CA19.9 level	1.17 (0.16, 8.46)	0.873	–	–
Clinical symptoms*	0.46 (0.06, 3.32)	0.441	–	–
**Grade of dysplasia**				
Low	1.00 (reference)		1.00 (reference)	
Intermediate	2.04 (0.98, 4.27)	0.058	1.94 (0.93, 4.07)	0.078
High	3.20 (1.51, 6.78)	0.002	2.96 (1.39, 6.29)	0.005
Invasive	9.41 (4.54, 19.50)	< 0.001	8.26 (3.95, 17.27)	< 0.001

Values in parentheses are 95% confidence intervals. *New onset or worsening of pre-existing diabetes mellitus, pancreatitis, or persisting abdominal symptoms. SPDP, spleen-preserving distal pancreatectomy; CA19.9, carbohydrate antigen 19.9.

## Discussion

In this first international cohort study of the role of splenectomy in patients undergoing distal pancreatectomy for IPMN, the LNM rate was 6.7% in the total cohort and 4.3% among patients without a preoperative suspicion of malignancy. Spleen-preserving distal pancreatectomy was associated with a shorter operating time, shorter hospital stay, and less blood loss than distal pancreatectomy with splenectomy, and comparable OS.

The 6.7% rate of LNM after distal pancreatectomy observed here cannot be compared with findings of previous studies as these combined all types of pancreatectomy for IPMN. In a single-year analysis^25^ in over 100 US centres, 21 patients (4.4%) had LNM among 478 patients after any type of pancreatectomy for IPMN. A single-centre series^26^ from Johns Hopkins identified 183 patients (29.7%) with malignancy and 97 (15.7%) with LNM among 616 patients undergoing any type of pancreatectomy for IPMN. Two smaller single-centre studies^27,28^ reported LNM in 7 of 98 (7%) and 27 of 244 (11.1%) patients undergoing any type of pancreatectomy for IPMN respectively.

The main benefit of spleen-preserving distal pancreatectomy is improvement in short-term outcome and preservation of splenic function, which may be considered especially important as most patients with IPMN have a very good life expectancy; however, the proportion of minimally invasive operations was higher in the spleen-preserving distal pancreatectomy group (*Table S6*), and so the results should be interpreted with caution. A 2014 meta-analysis^10^ compared outcomes after spleen-preserving distal pancreatectomy and distal pancreatectomy with splenectomy in 879 patients for all indications, and concluded that spleen-preserving distal pancreatectomy was associated with a shorter hospital stay (weighted mean difference 1.16, 95% c.i. −2.00 to −0.31; *P* = 0.007) and fewer intra-abdominal abscesses (OR 0.48, 0.27 to 0.83; *P* = 0.009), whereas other outcomes did not differ (such as blood loss and duration of operation). A more recent study^29^ reported high success rates (80%) for laparoscopic spleen-preserving distal pancreatectomy in 229 patients with benign and low-grade malignant disease, with no differences in postoperative morbidity in propensity score-matched patients, compared with 227 patients who underwent distal pancreatectomy with splenectomy. The authors concluded that spleen-preserving distal pancreatectomy is preferred for benign or low-grade malignant lesions owing to the increased risk of long-term complications after distal pancreatectomy with splenectomy. Another recent study^30^ of propensity score-matched patients (35 in each group) undergoing distal pancreatectomy for all indications found that the operating time was shorter for laparoscopic spleen-preserving distal pancreatectomy than for laparoscopic distal pancreatectomy with splenectomy. Furthermore, the authors noted higher quality-of-life (QoL) scores after spleen-preserving distal pancreatectomy, albeit the difference was not statistically significant. A follow-up study^31^ of 160 patients with benign or low-grade malignant disease reported improved QoL (less fatigue, symptoms of flu and cold, and better health condition) after spleen-preserving distal pancreatectomy *versus* distal pancreatectomy with splenectomy.

A possible disadvantage of spleen-preserving distal pancreatectomy is the risk of splenic infarction and splenic abscesses. Splenic infarction requiring reoperation was not observed in the present cohort, but other studies reported incidences ranging from 1.9 to 7.3%^31–33^. Long-term complications after spleen-preserving distal pancreatectomy according to Warshaw include left-sided portal hypertension and subsequent formation of epigastric varices. Unfortunately, these were not registered in the authors’ database and so it was not possible to provide data on this complication. Two of the aforementioned studies^31,32^ with long-term follow-up reported a 9 and 25% risk of varices after spleen-preserving distal pancreatectomy according to Warshaw in 65 and 111 patients respectively, although no significant gastrointestinal bleeding was observed.

Focusing on OS, the good life expectancy (90% after a median follow-up of 4.6 years) observed here is in accordance with a systematic review^9^ from 2016, in which the pooled 5-year survival rate in 2868 patients was 93.6% (95% c.i. 90.5 to 95.7). A more recently published abstract^34^ with 10-year nationwide follow-up of 88 resections (all types) for IPMN reported a 5-year survival rate of 87.5% for patients with low-grade dysplasia, 77.8% for those with high-grade dysplasia, and 35.9% for patients with invasive IPMN.

The present data suggest that spleen-preserving distal pancreatectomy was safe in patients with IPMN without preoperative suspicion of malignancy selected for this approach. According to the current policies for IPMN resection, most resected IPMNs do not harbour either high-grade dysplasia or invasive cancer^35,36^. Ultimately, a large pragmatic randomized trial should confirm the non-inferiority of spleen-preserving distal pancreatectomy to distal pancreatectomy with splenectomy for patients with IPMN without suspected malignancy. Such a study should include long-term follow-up to create insight into the long-term complications of both spleen-preserving distal pancreatectomy (for example varices) and distal pancreatectomy with splenectomy (such as OPSI), and should also include QoL questionnaires. In addition, standardization of lymph node station reporting is needed to distinguish LNMs accurately. If the results of these future studies show that spleen-preserving distal pancreatectomy has significant benefit over distal pancreatectomy with splenectomy in patients with IPMN, spleen-preserving distal pancreatectomy might be implemented in clinical practice, thus potentially improving surgical outcomes and QoL. In the present study, 5 of 106 patients had invasive cancer in the spleen-preserving distal pancreatectomy group but none underwent secondary splenectomy. The clinical consideration remains open for debate whether a secondary splenectomy should be performed.

The results of this study should be interpreted considering several limitations. First, owing to the retrospective design, the results are subject to indication bias. This is reflected by the higher dysplasia grade and higher rate of LNM in patients undergoing distal pancreatectomy with splenectomy for IPMN, indicating that patients with higher preoperative risk were specifically selected for distal pancreatectomy with splenectomy. Nevertheless, this was corrected for in Cox regression analyses. Second, it was not possible to provide detailed data on the location of LNMs (splenic hilum *versus* elsewhere) because this information was not present in most pathology reports. Additionally, stage migration might have taken place as a median of 11 (i.q.r. 5–20) lymph nodes were harvested per patient. Third, long-term follow-up was lacking in some patients, and the reliability of detection of the consequences of spleen-preserving distal pancreatectomy and distal pancreatectomy with splenectomy (for example OPSI) might therefore have been impaired. Fourth, data were not collected on IPMN recurrence. Fifth, the inclusion period of 15 years might have led to confounding because guidelines have changed over this interval, pancreatic cystic neoplasms are increasingly being diagnosed, and use of the minimally invasive approach has increased. The main strength of this study is its multicentre, international design with a considerable cohort of patients undergoing distal pancreatectomy for presumed IPMN. This study is also the first to provide insight into OS between patients undergoing spleen-preserving and those having distal pancreatectomy with splenectomy for IPMN. A future pragmatic randomized trial should confirm the non-inferiority of spleen-preserving distal pancreatectomy compared with distal pancreatectomy with splenectomy in patients requiring distal pancreatectomy for presumed IPMN without suspected malignancy.

## Supplementary Material

znad424_Supplementary_Data

## Data Availability

The data sets generated during and/or analysed during this study are not publicly available, but are available from the corresponding author on reasonable request.
