# Supplementary material for: Outcomes after distal pancreatectomy with or without splenectomy for intraductal papillary mucinous neoplasm: international multicentre cohort study
Source: Br J Surg. 2024 Jan 9;111(1):znad424. doi: 10.1093/bjs/znad424 (PMC10776207; doi:10.1093/bjs/znad424)
Supplement: znad424_Supplementary_Data [file znad424_supplementary_data.docx]

**SUPPLEMENTARY MATERIAL**

**Table S1. Collected variables**

|  | | | |
| --- | --- | --- | --- |
| **VARIABLE** | | **FORMAT** | |
| Date of form completion | | YYYY/MM/DD | |
| Hospital | | Drop-down | |
| Case ID | | Site name_pt_no | |
| Date of birth  **BASELINE – at time of surgery** | | YYYY/MM/DD | |
| Age in years | | YY | |
| Sex  ECOG performance status  ASA score  Co-morbidity?  If yes, please specify co-morbidity * | | M/F  0-4  1-4  Y/N  Cardiac/Vascular/Diabetes/Pulmonary/Neurological/Gastro-intestinal/Urogenital/Renal/Coagulopathic/Connective tissue disease/Immunologic/Oncologic/Other | |
| History of pancreatitis | | Y/N | |
| Level of CA 19.9 in serum | | U/mL | |
| **PRE-OPERATIVE DIAGNOSTICS** | |  | |
| Largest size of IPMN before surgery | | mm | |
| Dilatation of MPD | | Y/N | |
| If yes, diameter of MPD* | | mm | |
| Enhancing mural nodule | | Y/N | |
| If yes, size of mural nodule* | | mm | |
| Suspicion of solid mass | | Y/N | |
| Lymphadenopathy | | Y/N | |
| Cytology acquired? | | Y/N | |
| *Result of cytology | | Inconclusive/low-grade dysplasia / high-grade dysplasia / adenocarcinoma | |
|  | |  | |
| **INDICATION FOR RESECTION** | |  | |
| Indication for surgery (multiple answers possible) | | Positive cytology for HGD/Solid mass/Jaundice/Mural nodule/Thickened or enchancing cyst walls/Growth-rate/Cyst diameter/MPD dilatation/Abrupt change in MPD caliber with distal atrophy/Lymphadenopathy/Increased levels of serum CA19.9/New onset of diabetes mellitus/Worsening of diabetes mellitus/Pancreatitis/Other | |
| Pre-operative intention for splenectomy | | Y/N | |
|  | |  | |
| **PROCEDURE DETAILS** | |  | |
| Date of surgery  Date of hospital discharge | | YYYY/MM/DD  YYYY/MM/DD | |
| Type of resection | | Distal pancreatectomy including splenectomy/Spleen-preserving distal pancreatectomy | |
| If including splenectomy, reason*  If spleen-preserving, technique* | | Intention for splenectomy/peri-operatively decided on splenectomy due to anatomical reason  Splenic artery and vein preservation/Warshaw technique | |
| Operative time (incision to closure) | | Min. | |
| Intra-operative blood loss | | mL | |
| Surgical approach  Conversion minimally invasive to open | | Open/laparoscopic/robot-assisted/Other  Yes/No/Not applicable (procedure was primarily open) | |
|  | |  | |
| **PATHOLOGY** | |  | |
| Diagnosis | | Side-branch IPMN/Main-duct IPMN/Mixed-type IPMN/ Cancer/Other, namely…. | |
| Largest lesion size | | mm | |
| Grade of dysplasia | | Low grade/Intermediate grade/High grade/Invasive* | |
| *Overall resection margin of invasive component (Royal College of Pathologists definition) | | R0/R1/R2 | |
| * Final T*N*M* Stage (AJCC) | | T 0-4 /N 0-1 /M 0-1/Other | |
| *Grade of differentiation  Resection margin for IPMN  *Grade of dysplasia in resection plane | | Well differentiated/moderately differentiated/poorly differentiated  R0*/R1/R2  Low grade/Intermediate grade/High grade/Invasive | |
| Histopathological subtype | | Intestinal/Pancreatobiliary/Gastric/Oncocytic/Mixed | |
| Total number of harvested lymph nodes | | Count | |
| Number of malignant lymph nodes | | Count | |
| Station number of malignant lymph nodes  (more than one option possible) | | Not reported/1 - 18 | |
|  | |  | |
| **POSTOPERATIVE** |  | |  |
| Clavien-Dindo >3a complication within 90-days | | Y/N | |
| *Pancreatic fistula B/C within 90-days | | Y/N | |
| * Percutaneous drainage | | Y/N | |
| *EUS guided drainage | | Y/N | |
| *Date of removal surgical drain | | YYYY/MM/DD | |
| *Delayed gastric emptying B/C | | Y/N | |
| *Post-pancreatectomy hemorrhage B/C | | Y/N | |
| *Chyle leakage B/C | | Y/N | |
| Non-pancreas specific complications  Single organ failure?  Multi organ failure? | | Surgical site infection/Septic shock/Myocardial infarction/Cardiac arrest/Pulmonary embolism/Stroke  Y/N  Y/N | |
| Intensive Care Unit admission | | Y/N | |
| *Reason for ICU admission | | Free text | |
|  | |  | |
| **POST-DISCHARGE** | |  | |
| Death within 90-days post op | | Y/N | |
| *Date of death | | YYYY/MM/DD | |
| Readmission (related to index-procedure) within 90-days post op | | Y/N | |
| *Reason for re-admission | | Free text | |
| *Length of re-hospitalization | | DD | |
| **SURVIVAL AND MORBIDITY** | |  | |
| Date of last follow up | | YYYY/MM/DD | |
| Occurrence of secondary splenectomy | | Y/N | |
| New onset diabetes  New onset exocrine insufficiency  Occurrence of death | | Y/N  Y/N  Y/N | |
| Date of death* | | YYYY/MM/DD | |
| Abbreviations: AJCC = American Joint Committee on Cancer. ASA = American Society of Anesthesiologists. CA 19.9 = cancer antigen 19.9. DD= day. ECOG = Eastern Cooperative Oncology Group. F = female. HGD = high-grade dysplasia. ICU = intensive care unit. ID = identification. IPMN = intraductal papillary mucinous neoplasm. M = male. Min = minutes. mL = milliliter. MM = month. Mm = millimeter. MPD = main pancreatic duct. N = no. R = radicality. TNM = tumor-node-metastasis. U/mL = units per milliliter. Y= yes. YYYY = year. | | | |

**Table S2. Indications for resection in 700 patients after distal pancreatectomy for IPMN**

|  | **Total cohort**  **n = 700^a^** | **SPDP**  **n = 123** | **DPS**  **n = 577** | **p-value** |
| --- | --- | --- | --- | --- |
| Cytology ≥ HGD, n (%) | 72 (10.3) | 9 (7.3) | 63 (10.9) | 0.211 |
| Solid mass, n (%) | 102 (14.6) | 12 (9.8) | 90 (15.6) | 0.082 |
| Lymphadenopathy, n (%) | 7 (1.0) | 1 (0.8) | 6 (1.0) | > 0.99^b^ |
| Mural nodule, n (%) | 154 (22.0) | 17 (13.8) | 137 (23.7) | **0.012** |
| Thickened/enhanced cyst wall, n (%) | 44 (6.3) | 5 (4.1) | 39 (6.8) | 0.246 |
| MPD dilation, n (%) | 329 (47.0) | 51 (41.5) | 278 (48.2) | 0.123 |
| Abrupt change in MPD caliber, n (%) | 22 (3.1) | 3 (2.4) | 19 (3.3) | 0.781^b^ |
| Cyst diameter, n (%) | 254 (36.3) | 53 (43.1) | 201 (34.8) | 0.109 |
| Growth-rate, n (%) | 89 (12.7) | 20 (16.3) | 69 (12.0) | 0.219 |
| Elevated serum CA 19.9, n (%) | 71 (10.1) | 7 (5.7) | 64 (11.1) | 0.064 |
| Diabetes, n (%)  NODM  Worsening of existing DM | 2 (0.3)  4 (0.6) | -  - | 2 (0.3)  4 (0.7) | > 0.99^b^  > 0.99^b^ |
| Acute pancreatitis, n (%) | 35 (5.0) | 11 (8.9) | 24 (4.2) | **0.031** |
| Symptomatic cyst, n (%) | 15 (2.1) | 6 (4.9) | 9 (1.6) | **0.023** |
| Other, n (%) | 7 (1.0) | 4 (3.3)**^c^** | 3 (0.5)^d^ | **0.022**^b^ |
| Abbreviations: CA 19.9 = cancer antigen 19.9. DM = diabetes mellitus. DPS = distal pancreatectomy including splenectomy. HGD = high grade dysplasia. MPD = main pancreatic duct. N = number. NODM = new-onset diabetes mellitus. SPDP = spleen-preserving distal pancreatectomy.  ^a^  Indication for surgery was missing in 27 patients (4%); 3/123 SPDP patients (2%) and 24/577 DPS patients (4%).  ^b^ Fisher’s exact test was used.  ^c^ Indications for resection were: Young age (n = 2), family history of pancreatic cancer (n = 1), and presence of KRAS and GNAS mutations pancreatic cyst fluid (n = 1).  ^d^ Indications for resection were: Patient request (n = 2), and family history of pancreatic cancer (n = 1). | | | | |

**Table S3. Pathological outcomes and lymph node metastases in the per-protocol analysis of patients with preoperative suspected malignancy**

|  | SPDP  n = 14 | DPS  n = 120 | RR (95%CI) | p-value |
| --- | --- | --- | --- | --- |
| Grade of dysplasia, n (%)  Low  Intermediate  High  Invasive  Not reported | 5 (36)  1 (7)  2 (14)  4 (29)  2 (14) | 18 (15)  16 (13)  16 (13)  70 (58)  - | N/A | 0.104^a^ |
| Harvested lymph nodes, median (IQR) | 12 (2 – 21) | 17 (11 – 26) | N/A | 0.097 |
| Positive lymph nodes, n (%) | 1/14 (7) | 22/120 (18) | 0.39 (0.06 – 2.67) | 0.463^a^ |
| Percentages might not sum to 100% because of rounding.  Abbreviations: CI = confidence interval. DPS = distal pancreatectomy including splenectomy. IQR = interquartile range. N = number. N/A = not applicable. RR = relative risk. SPDP = spleen-preserving distal pancreatectomy.  ^a^ Based on Fisher’s exact test. | | | | |

**Table S4. Pathological outcomes and lymph node metastases in the intention-to-treat analysis of patients with preoperative suspected malignancy**

|  | SPDP  n = 17 | DPS  n = 117 | RR (95%CI) | p-value |
| --- | --- | --- | --- | --- |
| Grade of dysplasia, n (%)  Low  Intermediate  High  Invasive  Not reported | 6 (35)  1 (6)  3 (18)  7 (41)  - | 17 (15)  16 (14)  15 (13)  67 (57)  2 (1.7) | N/A | 0.16^a^ |
| Harvested lymph nodes, median (IQR) | 15 (5 – 26) | 17 (11 – 26) | N/A | 0.47 |
| Positive lymph nodes, n (%) | 2/17 (12) | 21/117 (18) | 0.66 (0.17 – 2.55) | 0.74^a^ |
| Percentages might not sum to 100% because of rounding.  Abbreviations: CI = confidence interval. DPS = distal pancreatectomy including splenectomy. IQR = interquartile range. N = number. N/A = not applicable. RR = relative risk. SPDP = spleen-preserving distal pancreatectomy.  ^a^ Based on Fisher’s exact test. | | | | |

**Table S5. Pathological outcomes and lymph node metastases in the intention-to-treat analysis of patients without preoperative suspected malignancy**

|  | SPDP  n = 122 | DPS  n = 417 | RR (95%CI) | p-value |
| --- | --- | --- | --- | --- |
| Grade of dysplasia, n (%)  Low  Intermediate  High  Invasive  Not reported | 63 (52)  27 (22)  16 (13)  8 (7)  8 (7) | 144 (35)  120 (290)  84 (20)  53 (13)  16 (4) | N/A | **0.002** |
| Harvested lymph nodes, median (IQR) | 4 (1 – 9) | 12 (6 – 20) | N/A | **< 0.001** |
| Positive lymph nodes, n (%) | 3/122 (3) | 20/417 (5) | 0.51 (0.16 – 1.70) | 0.32^a^ |
| Nodule or enhancing wall | 2/21 (10) | 4/95 (4) | 2.26 (0.44 – 11.55) | 0.297^a^ |
| Main duct involvement | 0/47 (0) | 10/180 (6) | N/A | 0.127^a^ |
| Growth or cyst size | 1/43 (2) | 4/97 (4) | 0.55 (0.06 – 4.79) | > 0.99^a^ |
| Increased CA 19.9 | 0/1 (0) | 1/6 (17) | N/A | > 0.99^a^ |
| Clinical symptoms^b^ | 0/6 (0) | 0/4 (0) | N/A | N/A |
| Other indication | 0/3 (0) | 1/11 (9) | N/A | > 0.99^a^ |
| Percentages might not sum to 100% because of rounding.  Abbreviations: CA 19.9 = cancer antigen 19.9. CI = confidence interval. DPS = distal pancreatectomy including splenectomy. IQR = interquartile range. N = number. N/A = not applicable. RR = relative risk. SPDP = spleen-preserving distal pancreatectomy.  ^a^ Based on Fisher’s exact test.  ^b^ New onset or worsening of preexisting diabetes mellitus, pancreatitis, or persisting abdominal symptoms were classified as clinical symptoms. | | | | |

**Table S6. Surgery and pathology characteristics after distal pancreatectomy for IPMN**

|  | **SPDP**  **n = 123** | **DPS**  **n = 577** | **p-value** |
| --- | --- | --- | --- |
| Surgical characteristics | | | |
| Procedure time in min (median, IQR)^a^ | 180 (120 – 241) | 226 (162 – 280) | **0.001** |
| EBL in mL (median, IQR)^b^ | 100 (50 – 250) | 336 (100 – 383) | **0.001** |
| Surgical approach, n (%)  Open  Laparoscopic  Robot-assisted | 64 (52)  51 (41)  8 (7) | 415 (72)  136 (24)  26 (5) | **< 0.001** |
| Adverse events (CD ≥3), n (%)  POPF  DGE  Hemorrhage  Chyle leakage  Non-pancreatic complications | 14 (11)  13 (11)  2 (2)  3 (2)  3 (2)  2 (2)^c^ | 58 (10)  57 (10)  8 (1)  13 (2)  2 (0.3)  21 (4)^d^ | 0.66 |
| Pathological characteristics | | | |
| Final pathological diagnosis, n (%)  Side-branch IPMN  Main-duct IPMN  Mixed-type IPMN  Cancer  IPMN NOS  Other  Not reported | 43 (35) 20 (16) 40 (33) 3 (2)  6 (5) 8 (7)^e^ 3 (2) | 152 (26)  105 (18)  198 (34)  71 (12)  20 (3)  6 (1)^f^  25 (4) | **< 0.001**^g^ |
| Histopathological subtype, n (%)  Intestinal  Pancreatobiliary  Gastric  Oncocytic  Mixed  Not reported | 12 (10)  2 (2)  60 (49)  2 (2)  9 (7)  38 (31) | 62 (11)  17 (3)  197 (34)  12 (2)  27 (5)  262 (45) | **0.009**^g^ |
| Long-term outcomes | | | |
| New-onset diabetes mellitus, n (%) | 28 (23) | 147 (26) | 0.24 |
| Exocrine pancreatic insufficiency, n (%) | 18 (15) | 96 (17) | 0.51 |
| Percentages might not sum up to 100% because of rounding.  Abbreviations: CD = Clavien-Dindo score. DPS = distal pancreatectomy including splenectomy. EBL = estimated blood loss. IQR = interquartile range. N = number. SPDP = spleen-preserving distal pancreatectomy.  ^a^ Procedure time was missing in 9 SPDP patients (7%) and 52 DPS patients (9%).  ^b^ EBL was missing in 7 SPDP patients (6%) and 99 DPS patients (17%).  ^c^ One myocardial infarction and one cardiac arrest.  ^d^ Non-pancreatic complications consisted of: surgical site infection (n = 9), septic shock (n = 2), myocardial infarction (n = 6), liver failure (n = 1), urinary retention (n = 1), thromboembolic event (n = 2).  ^e^ Other diagnosis included: pseudocyst (n = 2), retention cyst (n = 2), pancreatic stricture (n = 1), no abnormalities (n = 1), MCN (n = 1), and a benign cyst (n = 1).  ^f^ Other diagnosis included: pseudocyst (n = 2), SCN (n = 1), pNET (n = 1), pancreatic cyst NOS (n = 1), and intraductal papillary myxoma (n = 1).  ^g^ Based on Fisher’s exact test. | | | |

**
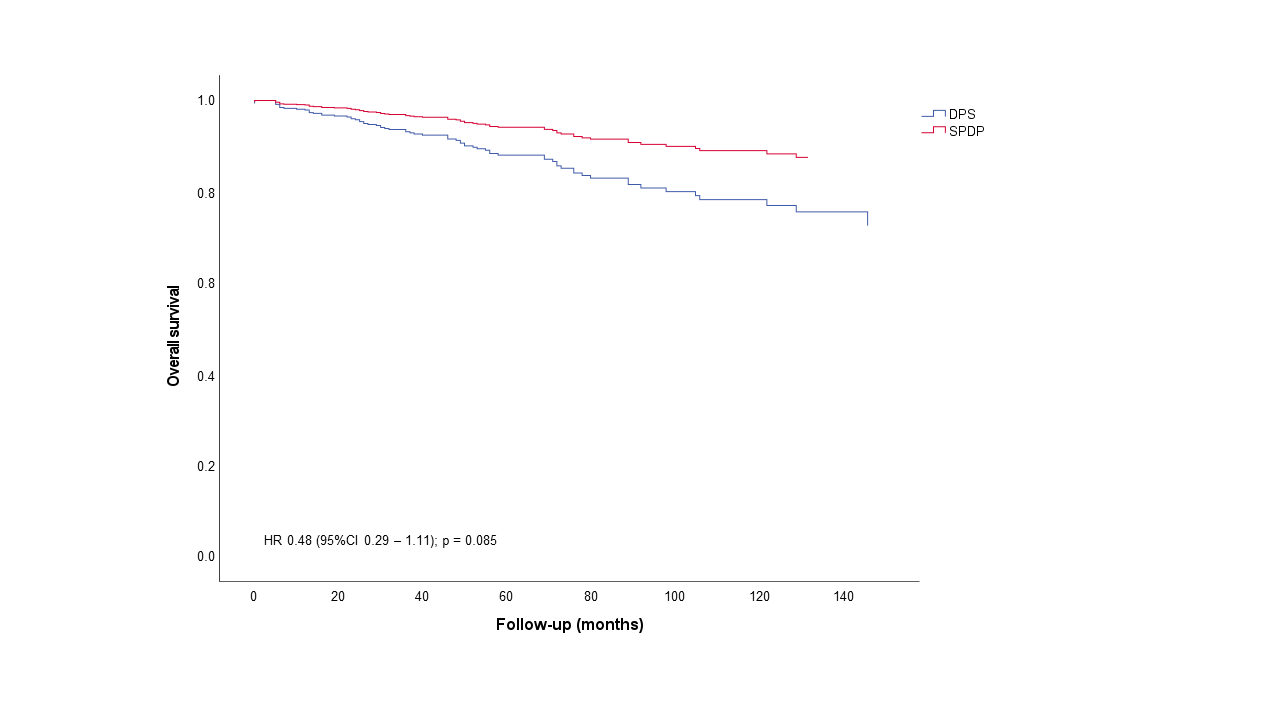
Figure S1. Estimates of overall survival after SPDP and DPS in patients without preoperative suspected malignancy, corrected for dysplasia grade**

Abbreviations: CI = confidence interval. DPS = distal pancreatectomy including splenectomy. HR = hazard ratio. SPDP = spleen-preserving distal pancreatectomy.
